# Effects of Leader–Member Exchange Quality on the Internalization of Emotional Regulation: The Moderating Effect of Mentoring Functions

**DOI:** 10.1155/jonm/4772084

**Published:** 2026-04-06

**Authors:** Li-Chuan Chu

**Affiliations:** ^1^ Department of Health Policy and Management, Chung Shan Medical University, Taichung, Taiwan (ROC), csmu.edu.tw; ^2^ Department of Medical Education, Chung Shan Medical University Hospital, Taichung, Taiwan (ROC), csh.org.tw

**Keywords:** internalization of emotional regulation, leader–member exchanges quality, mentoring functions

## Abstract

**Background:**

Research has indicated that the internalization of emotion regulation is an important process for promoting positive work outcomes among employees. In Taiwan nursing contexts, however, the collectivist culture and high power distance within the Chinese community, along with the hierarchical and highly structured nature of nursing work, are not conducive to the internalization of emotional regulation. This study was focused on exploring mechanisms or policies within hospitals that can improve the internalization of emotional regulation among nursing staff. This study examined how the quality of leader–member exchange (LMX) influences the internalization of emotional regulation and explored the moderating role of mentorship in this relationship.

**Methods:**

A two‐stage questionnaire survey was conducted to limit common method bias. Female nurses in a medical center in central Taiwan participated. The recruitment period for this study started in April 2020 and ended in June 2020. A total of 300 questionnaires were distributed at each stage, and 252 matched pairs of responses were retrieved (valid response rate = 84%). The proposed hypotheses were verified using hierarchical regression conducted with SPSS Version 25.0.

**Results:**

The findings suggest that LMX quality positively influences the internalization of emotional regulation (*β* = 0.26, *p* < 0.01). Additionally, the results confirm that mentoring functions strengthen this positive relationship (*β* = 0.18, *p* < 0.01), highlighting their role as a moderating factor.

**Conclusions:**

This study confirmed that establishing high‐quality LMX relationships can enhance the internalization of emotional regulation among female nursing staff. In addition, mentors can provide effective guidance and feedback, thereby improving the quality of LMX and their internalization of emotional regulation. Strengthening LMX relationships and establishing a comprehensive mentoring system are important for hospitals.

## 1. Introduction

When individuals’ emotion regulation behaviors are increasingly aligned with their personal values and beliefs—that is, when emotional regulation is internalized—such internalization may contribute to enhanced well‐being [1] and improved job performance [2]. However, the internalization process is strongly influenced by cultural context. In Chinese communities, characterized by collectivism and high power distance, individuals are often expected to maintain interpersonal harmony and adhere to role norms, which may inhibit the expression of negative emotions [3].

This phenomenon may be particularly pronounced in healthcare settings, where organizations feature clear hierarchical structures and strict professional standards, and nurses face heavy workloads while responding to multiple emotional and institutional demands [4]. In such high‐pressure and emotionally demanding environments, nurses may be more inclined to adopt suppressive or externally controlled forms of emotion regulation, thereby increasing the risk of burnout [5] and diminishing well‐being [6]. Consequently, identifying effective organizational strategies to facilitate the internalization of emotional regulation in high‐pressure clinical environments remains a critical theoretical and practical challenge.

An increasing body of research suggests that high‐quality leader–member exchange (LMX) can enhance employees’ autonomous motivation by satisfying basic psychological needs [7, 8]. This process may support the internalization of emotional regulation among nurses. However, few studies have examined LMX as a contextual resource for promoting emotional regulation internalization from a self‐determination theory (SDT) perspective.

Moreover, in clinical settings, clinical teachers serve not only as professional guides but also as important sources of psychological support for nurses [9, 10]. The support mechanisms established through mentorship may further strengthen the positive impact of high‐quality LMX on the internalization of emotional regulation. Yet, empirical evidence regarding the moderating role of mentorship in the relationship between LMX and emotional regulation internalization remains limited. Therefore, this study examines whether mentorship moderates and reinforces the positive association between LMX and nurses’ internalization of emotional regulation.

In summary, this study integrates SDT, LMX theory, and mentorship perspectives to investigate the influence of LMX quality on the internalization of emotional regulation among nurses within Taiwan’s healthcare context and examine the moderating role of mentorship. This integrative framework not only addresses theoretical gaps in understanding the mechanisms of emotional regulation internalization but also deepens insight into the internalization of motivation within cultural contexts and provides empirical evidence for human resource and emotional support strategies in healthcare organizations.

### 1.1. Effects of LMX Quality on the Internalization of Emotional Regulation

In the internalization of emotional regulation model, when people do not express emotions, their intentions are spontaneous nonexpression or nonexpression due to forced regulation. The nonexpression of emotions has various effects on physical and psychological health [11]. According to SDT [12, 13], intentional behavior can be broadly classified as controlled or autonomous, depending on whether it is driven by interpersonal or intrapsychic pressure (controlled behavior) or regulated through personal choice (autonomous behavior). Within this framework, internalization refers to the process through which external norms or demands are taken in and regulated by the self. Importantly, SDT further distinguishes different forms of internalization, which give rise to qualitatively different types of emotional regulation. Accordingly, emotional regulation can be categorized into four types based on the degree of internalization: external regulation, introjected regulation, identified regulation, and integrated regulation [11, 14].

External regulation is a regulation that occurs completely because of being ordered. Introjected regulation involves forcing oneself to perform certain behavior in order to comply with expectations that they identify with. In essence, introjected regulation is closely attached to external forces. People often suppress the expression of negative emotions because they think they will feel ashamed or guilty if they do not. Both external and introjected regulations represent controlled behavior and are less spontaneous. When one agrees that regulation is valuable, the resulting regulation behavior is identified regulation. For example, members of a group may naturally suppress negative emotions to avoid destroying an atmosphere of group harmony. Finally, when internalization is complete, one integrates the aforementioned identification into other aspects of oneself and displays greater autonomy in one’s behavior; this is known as integrated regulation. For example, when individuals are aware of emotions, they completely regulate their emotional expression of their own will. Such regulatory behavior is completely spontaneous rather than an act of compliance with social norms. In the case where the emotional regulation has been integrated, an individual experiences little internal conflict. Compared with external and introjected regulation, identified and integrated regulation is regarded as spontaneous internalization behavior [11]. Subsequent studies have also verified that autonomous emotional regulation (either identified or integrated regulation) is beneficial to physical and mental health [1, 15]. Therefore, hospitals should consider how to effectively promote the internalization of emotional regulation among nursing staff. The interactive relationships between employees and supervisors within an organization may play a crucial role in this process.

LMX theory suggests that because supervisors have limited resources (time, energy, or openings for promotion), they establish interactive relationships of varying degrees with their subordinates. Subordinates who are competent, cooperative, and extroverted are more likely to be included in the supervisor’s inner circle and have closer relationships with them [16, 17]. In a high‐quality LMX, subordinates and supervisors have close interactions, and the mutual trust and respect between them result in subordinates typically enjoying more opportunities, resources, and guidance [18, 19]. By contrast, in low‐quality LMX relationships, employees lack emotional exchanges with supervisors and do not receive support or opportunities for development, and typically only engage in interactions and follow regulations as required by the job [20].

Previous research has confirmed that high‐quality LMX relationships have numerous positive effects on employees, effectively reducing their emotional exhaustion [21] and job stress [22], while helping to improve their physical and psychological health [23]. Employees are more willing to engage in their work [24] and are more likely to have favorable job performance [8], experience a stronger sense of belonging to the organization [25], and have lower turnover intentions [26].

Glasø and Einarsen [27] demonstrated that high‐quality LMX relationships between supervisors and subordinates involve less emotional regulation, suppression, and dissembling. Xu et al. [28] uncovered a negative correlation between emotional masking—the expression of insincere emotions—in subordinates and LMX relationships between supervisors and subordinates. Medler‐Liraz and Seger‐Guttmann [29] verified a positive correlation between high‐quality LMX and employees’ authentic positive emotional displays. During interpersonal interactions, emotional masking hinders mutual understanding and adversely affects the establishment of close relationships [30]. Subordinate emotional expression is an integral component of leader–subordinate communications. Without effective emotional exchanges, mutual understanding will become problematic and hinder the formation of good will, trust, and commitment in interpersonal relationships [28]. That is, in high‐quality exchange relationships between supervisors and subordinates, the subordinate’s emotional regulation may appear as the employee being more genuine and sincere and less inhibited or inauthentic.

In SDT [31], individuals strengthen their autonomous motivations through the satisfaction of three needs, namely competence, autonomy, and relatedness. In high‐quality LMX, employees receive attention, trust, work support, and resource provision from supervisors [19]; these advantages help improve job competencies [19], satisfying the “competence” requirement. Trust also creates more room for autonomy; employees are given more opportunities to participate in decision‐making processes, satisfying the “autonomy” requirement [32]. In these interactions, employees receive emotional support from their supervisors, gain access to the supervisor’s social network, and establish connections with other professionals, satisfying the “relatedness” requirement [32].

The satisfaction of these psychological needs can enhance the autonomous motivation of employees [7], enabling their behavior to better align with their own values, interests, and goals. Employees who establish higher quality interactive relationships with their supervisors are more likely to be autonomously motivated [7, 8]. Autonomous motivations encourage individuals to engage in autonomous emotional regulation when suppressing negative emotions [33]. By contrast, when employees’ needs are unmet, they may feel coerced into the behaviors they perform. In other words, within a high‐quality LMX, subordinates may experience a higher degree of emotional regulation internalization, enabling them to regulate their emotions proactively based on personal values and motivations. Therefore, in situations that require emotional suppression, they may choose to suppress negative emotions spontaneously and voluntarily, driven by internal self‐regulation, rather than being influenced by external pressures.

Based on the above discussion, this study posited the following hypothesis:

• Hypothesis 1: LMX quality positively influences the internalization of emotional regulation.

### 1.2. The Moderating Effect of Mentoring Functions

Mentorship is the interpersonal relationship between a senior staff member (mentor) and a junior staff member (apprentice). Mentors provide apprentices with career development functions, psychosocial functions [34], and role modeling [35]. Career development functions include the mentor acting as a coach, sharing professional knowledge and skills, guiding the apprentice’s learning, assigning the apprentice challenging tasks, providing feedback, introducing the strengths of the apprentice to others, increasing the apprentice’s exposure, helping the apprentice establish relationships and a reputation, and protecting the apprentice whenever appropriate. Psychosocial functions mean that the mentor has confidence in, approves, encourages, and supports the apprentice’s performance, plays a consultant role, shares his or her experience, and cares for and establishes a friendship with the apprentice. In role modeling, the mentor intentionally or unintentionally establishes an ideal model for the apprentice, who learns the working methods, attitudes, and values of the mentor [36].

Mentorship can be formal or informal [37]. In formal mentorship, the organization generally determines the matching of mentors and apprentices and the forms of interaction between them. Informal mentorship is an informal relationship formed voluntarily by the mentor and apprentice and is usually based on psychological or work development needs. The nature of the mentorship influences interaction quality and the number of mentoring functions [38]. When the mentor is a direct supervisor, the apprentice obtains more career development functions. By contrast, when the mentor is not a direct supervisor, the apprentice receives more psychosocial functions [39].

How can mentorship in nursing promote LMX quality? In hospitals, a head nurse (supervisor) assigns trained senior nurses as clinical teachers (mentors) for new nurses (apprentices) [40]. Through one‐on‐one guidance and assistance, clinical teachers demonstrate nursing skills and work attitudes, help new nurses adapt to the workplace [41], improve professional expertise [9], enhance identification with the hospital [42] and self‐efficacy [10], alleviate job stress, and reduce nurses’ turnover intentions [43]. These improvements allow new nurses to perform to a higher standard at work and demonstrate positive work attitudes; such nurses are more likely to win the head nurse’s recognition and trust, improving the quality of the LMX relationship [16].

In summary, the author believed that compared with employees who received poor mentoring functions, those receiving more satisfactory mentoring functions share a better relationship quality with their supervisors and thus possess a higher level of internalization of emotional regulation. Restated, mentoring functions may effectively moderate the positive relationship between LMX and the internalization of emotional regulation.

Based on the above discussion, this study posited the following hypothesis:

• Hypothesis 2: Mentoring functions moderate the positive relationship between LMX and the internalization of emotional regulation; more satisfactory mentoring strengthens the positive relationship between LMX and the internalization of emotional regulation.

**FIGURE 1:**
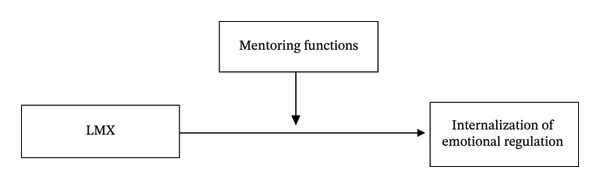
Research framework.

The research framework is presented in Figure 1.

## 2. Materials and Methods

### 2.1. Study Design and Participants

To avoid common method bias [44], a two‐stage questionnaire survey was adopted. The first primarily measured the LMX, mentoring functions, and demographic variables of the participants. After 2 months, the same participants were asked to complete a self‐assessment questionnaire, which included items on the internalization of emotional regulation. Female nurses in a medical center in central Taiwan were recruited. Data collection was conducted from April 2020 to June 2020. A total of 300 questionnaires were distributed in the first stage in convenience sampling. Again, the second stage of the survey was conducted 2 months afterward. In the two‐stage questionnaire method, distributors of the questionnaires provided codes or symbols that they understood on the upper right corner of Questionnaires A and B for the same participant to facilitate matching of the questionnaires.

Through the two‐stage distribution, after the exclusion of participants who had not received guidance from clinical teachers and invalid responses, 256 questionnaires were initially collected. To ensure the robustness of the statistical model and multivariate normality, the Mahalanobis distance was calculated for all core variables [45]. Based on the critical value (*χ* ^2^ = 22.46, df = 6, *p* < 0.001), 4 surveys were identified as multivariate outliers and excluded from the study. Consequently, 252 valid questionnaires were retained for the final analysis, yielding an 84.0% valid response rate. In terms of marital status, 201 participants (79.8%) were unmarried. As regards educational attainment, 210 (83.3%) had a university degree. Regarding service unit department, 160 (63.5%) worked in the ward. Their average age was 29.40 years, and their average tenure at their current hospital was 6.28 years. The result is presented in Table 1.

**TABLE 1:** Sociodemographic characteristics of sample (*n* = 252).

Characteristics/categories	*N*	%
Marital status		
Single	201	79.8
Married	51	20.2
Education level		
Associate degree	28	11.1
Bachelor’s degree	210	83.3
Graduate school and above	14	5.6
Department		
Intensive care unit	86	34.1
Ward	160	63.5
Others	6	2.4

	**Mean**	**SD**

Age	29.40	6.71
Seniority	6.28	6.07

### 2.2. Measures

LMX quality was measured using the LMX scale developed by Liden and Maslyn [46], which has 11 items. Subordinates were asked to evaluate the quality of their relationship with their supervisors. An example is “I admire the professional expertise of my supervisor.” A 6‐point Likert scale was used ranging from 1 (“*strongly disagree*”) to 6 (“*strongly agree*”), with a higher score indicating higher LMX quality. Previous research has reported satisfactory reliability (Cronbach’s *α* = 0.82) and criterion‐related validity for this scale [47]; meanwhile, the scale in the present study exhibited excellent internal consistency (Cronbach’s *α* = 0.95).

Mentoring functions was measured using the mentoring functions scale developed by Castro and Scandura [48], which contains a total of nine items on career support, psychosocial functions, and role modeling functions (e.g., “My mentor will devote his or her extra time and effort to my career.”). Subordinates were asked to evaluate the mentoring functions provided specifically by their assigned mentor (clinical teachers). The scale was scored using a 6‐point Likert scale (1 “*strongly disagree*” to 6 “*strongly agree*”), with a higher score indicating that the nurse perceived the mentor as providing more, stronger, and more comprehensive functions. Previous research has reported high reliability (Cronbach’s *α* = 0.93) and satisfactory criterion‐related validity for this scale [49]; meanwhile, the scale in the present study exhibited excellent internal consistency (Cronbach’s *α* = 0.95).

Internalization of emotional regulation was measured using the Self‐Regulation of Withholding Negative Emotions Questionnaire developed by Kim et al. [11]. The scale primarily measures the level of self‐regulation of negative emotion suppression and contains four subdimensions, namely external regulation, introjected regulation, identified regulation, and integrated regulation. Identified and integrated regulations (e.g., “It is important to remain calm”) are regarded as spontaneous internalization behaviors [11]. Therefore, the author integrated the two subdimensions as the internalization of emotional regulation, which consisted of a total of seven items. Responses again ranged from 1 (“*strongly disagree*”) to 6 (“*strongly agree*”), with a higher score implying a higher level of internalization of emotional regulation. Consistent with the satisfactory reliabilities reported in previous research for the identified and integrated (Cronbach’s *α* = 0.72 and 0.76, respectively) subscales [15], the combined 13‐item internalization scale in the present study demonstrated excellent internal consistency (Cronbach’s *α* = 0.93).

Variables that might affect the internalization of emotional regulation were selected as control variables. First, nursing seniority was included, as nurses with greater seniority typically have more work experience; thus, they are likely to have higher autonomy [50] and possess stronger senses of competence [51] and belonging at work [52], which relate to the three basic psychological needs of autonomy, competence, and relatedness. Consequently, these nurses might possess higher autonomous motivation [53], which in turn would increase the likelihood of the internalization of emotional regulation [33].

Second, satisfaction with clinical teachers was included as a control variable, as overall teaching satisfaction may influence participants’ evaluations of mentoring functions and LMX. According to the halo effect [54], an individual’s general affective evaluation could permeate their judgments of specific interpersonal interactions and psychological processes, potentially leading to evaluation bias [55]. Furthermore, satisfaction with clinical teachers reflects a positive affective state within the learning environment [56], which could inherently facilitate the internalization of emotion regulation [33, 57]. Therefore, to exclude potential confounding effects, this study controlled for satisfaction with clinical teachers in the analysis to ensure that the influences of LMX and mentoring functions on emotion regulation internalization possess relatively independent explanatory power.

### 2.3. Ethical Considerations

Before the questionnaires are distributed, we have obtained department leaders’ consent in assisting questionnaire distribution. All participants provided written informed consent before completing the survey. They were informed about the study’s purpose and were assured of their right to withdraw at any time. Their survey data were handled confidentially. In accordance with the Ethical Considerations, the protocol for this research was approved by the Institutional Review Board at Taiwan (IRB No.: CS19063).

### 2.4. Data Analysis

The questionnaire data collected in this study were analyzed using SPSS Version 25.0, a widely recognized software in academic research due to its robust statistical tools and ability to handle complex multivariate data efficiently. Descriptive statistics were employed to assess the demographic characteristics of the nurses. A correlation analysis was employed to explore the relationships between variables. Hierarchical regression analysis was conducted to test the hypotheses of this study. The level of statistical significance was set at *p* < 0.05.

## 3. Results

### 3.1. Reliability and Validity

Confirmatory factor analysis (CFA) was conducted using JASP 0.95.4.0 to evaluate the measurement model and verify the psychometric properties of the scales based on the final sample of *N* = 252. The measurement model, consisting of the latent constructs of LMX, mentoring functions, and internalization of emotional regulation, demonstrated an excellent fit to the data: CFI = 1.00, TLI = 1.22, RMSEA < 0.001, and SRMR = 0.054. Internal consistency was confirmed through Cronbach’s alpha (*α*) ranging from 0.93 to 0.95 and McDonald’s omega (ω) ranging from 0.92 to 0.94, all well above the recommended 0.70 threshold. Convergent validity was supported as all standardized factor loadings were significant (*p* < 0.001) and ranged from 0.61 to 0.93 following the exclusion of one item from the LMX scale and one item from the internalization of emotional regulation scale to improve model parsimony. Furthermore, the average variance extracted (AVE) for LMX (0.72), mentoring functions (0.77), and internalization of emotional regulation (0.57) all exceeded the 0.50 benchmark. The analysis results revealed that the measurement model has convergent validity and good reliability.

### 3.2. Descriptive Statistics of Major Variables

According to the correlation analysis results in Table 2, LMX quality, mentoring functions, and internalization of emotional regulation shared a significant correlation. Specifically, LMX quality was significantly positively correlated with mentoring functions (*r* = 0.47, *p* < 0.01) and internalization of emotional regulation (*r* = 0.33, *p* < 0.01), and mentoring functions were significantly positively correlated with internalization of emotional regulation (*r* = 0.42, *p* < 0.01). To ensure that the moderate correlation between LMX and mentoring functions (*r* = 0.47) did not pose a multicollinearity threat, VIF diagnostics were performed. The results showed that VIF values for all predictors ranged from 1.01 to 1.69, well below the conservative threshold of 3.0, indicating that these constructs are statistically distinct for regression analysis.

**TABLE 2:** Descriptive statistics and intercorrelations among study variables.

Variable	1	2	3
1. LMX quality	(0.95)		
2. Mentoring functions	0.47^∗∗^	(0.95)	
3. Internalization of emotional regulation	0.33^∗∗^	0.42^∗∗^	(0.93)
Mean	4.99	4.78	4.63
SD	0.61	0.78	0.65

*Note:* Cronbach’s alphas appear on the diagonal.

^∗^ *p* < 0.05.

^∗∗^ *p* < 0.01.

### 3.3. Hypothesis Testing

**FIGURE 2:**
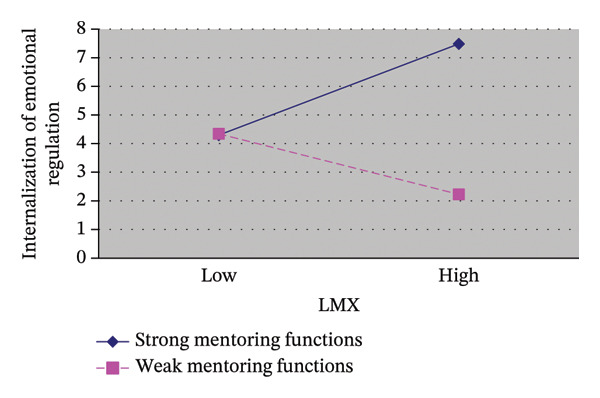
Plot of the interaction between LMX quality and mentoring functions on the internalization of emotional regulation.

According to the hierarchical regression analysis results in Table 3, control variables such as satisfaction with clinical teacher teaching was a significant positive predictor of the internalization of emotional regulation (*β* = 0.30, *p* < 0.01). After controlling for factors (e.g., seniority, satisfaction with teaching) that might affect the dependent variables, LMX quality was found to significantly and positively predict the internalization of emotional regulation (*β* = 0.26, *p* < 0.01). Thus, the first hypothesis (H1) was supported. When mentoring functions were added to the model (Step 3), it was a significant positive predictor of the internalization of emotional regulation (*β* = 0.35, *p* < 0.01). The interaction between LMX quality and mentoring functions significantly positively predicted the internalization of emotional regulation (*β* = 0.18, *p* < 0.01). The differential effect of high and low mentoring functions on the relationship between LMX and internalization of emotional regulation is shown in Figure 2. In the group with strong mentoring functions, LMX quality displayed a significant positive correlation with the internalization of emotional regulation (*β* = 0.52, *p* < 0.01). In the group with weak mentoring functions, LMX quality exhibited a slight positive correlation with the internalization of emotional regulation (*β* = −0.30, *p* > 0.05) that was nonsignificant. In brief, with strong mentoring functions, the positive relationship between LMX quality and the internalization of emotional regulation was strengthened. By contrast, with weak mentoring functions, the correlation was weaker and nonsignificant. Accordingly, the second hypothesis (H2) was partially supported.

**TABLE 3:** Results of regression analyses on the internalization of emotional regulation: the moderating effect of mentoring functions

Independent variables	Dependent variables			
	Internalization of emotional regulation			
	Step 1	Step 2	Step 3	Step 4
	Beta	Beta	Beta	Beta
Seniority	0.12	0.09	0.12	0.12^∗^
Satisfaction with the teaching of clinical teachers	0.30^∗∗^	0.21^∗∗^	0.05	0.05
z LMX quality		0.26^∗∗^	0.15^∗^	0.13^∗^
z Mentoring functions			0.35^∗∗^	0.36^∗∗^
z LMX quality × z mentoring functions				0.18^∗∗^
*R* ^2^	0.08	0.14	0.21	0.24
Adjusted *R* ^2^	0.07	0.13	0.19	0.23
△*R* ^2^	0.08^∗∗^	0.06^∗∗^	0.07^∗∗^	0.03^∗∗^
*F*	10.90	13.44	16.13	15.58

*Note: N* = 252.

^∗^ *p* < 0.05.

^∗∗^ *p* < 0.01.

## 4. Discussion

This study explored the influence of LMX quality on the internalization of emotional regulation among nursing staff. Additionally, mentoring was examined as a moderating variable to determine whether it strengthens the positive relationship between LMX quality and the internalization of emotional regulation.

The research results indicate that LMX quality was a significant positive predictor of the internalization of emotional regulation. Employees who perceive a high‐quality exchange relationship with their supervisors are more likely to internalize emotional regulation, adopting identified or integrated regulation when managing negative emotions. This finding provides empirical evidence of the positive association between LMX quality and emotional regulation internalization, contributing to a better understanding of how leader–member relationships may be linked to employees’ willingness to regulate emotions in ways consistent with their personal values. Based on LMX theory and SDT, employees in a high‐quality exchange relationship receive greater trust, support, and resources from their supervisors, reinforcing their sense of competence, autonomy, and connection within the organization [7, 8]. These benefits facilitate the internalization of emotional regulation, enabling employees to regulate their emotions more autonomously and spontaneously, rather than merely conforming to external pressures [11, 33]. This study extends prior research by demonstrating that LMX quality itself can serve as a key factor in promoting emotional regulation internalization, highlighting its importance in workplace emotional management.

The present study also verified that mentoring functions moderate the positive relationship between LMX quality and the internalization of emotional regulation. In particular, when hospitals implement rigorous mentorship, the positive relationship between LMX quality and the internalization of emotional regulation is strengthened. This finding extends LMX theory and emotional regulation theory by revealing the key moderating role of mentoring. When mentors are guiding their apprentices, they provide the apprentices with career development and psychosocial support, acting as a role model for the apprentices to learn from and helping apprentices improve their professional competence [9]. Such growth and improvements increase their chances of being included in the supervisor’s inner circle [16], enhancing the quality of exchange relationships between the supervisor and their subordinates and promoting the internalization of emotional regulation.

Regarding control variables, the findings reveal that nurses with longer tenure exhibit higher levels of internalization of emotion regulation. This may be attributed to the fact that senior nurses typically possess extensive clinical experience, which provides them with greater autonomy and a stronger sense of competence and relatedness within the workplace [50–52]. According to SDT, when these basic psychological needs are satisfied, individuals develop higher autonomous motivation [53], which in turn enhances the internalization of emotion regulation [33]. This suggests that years of clinical practice may not only cultivate professional expertise but could also potentially facilitate nurses’ internal growth in emotional regulation.

In addition, the findings indicate that nurses who were more satisfied with their clinical teachers reported increased internalization of emotional regulation. When the nurses were satisfied with their clinical teachers, they reported higher organizational commitment, work engagement, job satisfaction, and social support [58]. Satisfactory learning experiences enabled them to experience a sense of competence, feel connected to others, and satisfy their needs for feeling competent and related. Therefore, they had higher autonomous motivation [53], which increased their internalization of emotional regulation.

### 4.1. Limitations

This study had several limitations. First, to reduce common method bias, a two‐stage questionnaire survey was conducted; however, the independent and moderating variables were simultaneously self‐assessed by the participants. Thus, the possibility of bias due to the common method effect exists. The author recommends that future studies adopt a three‐stage test or the measurement of variables by other participants (e.g., supervisors or peers). Second, the study was focused on the overall effectiveness of mentoring functions in their moderating effect on the relationship between LMX quality and the internalization of emotional regulation. Future studies may explore differences in the effects of various indicators of mentoring functions (e.g., career development functions, psychosocial functions, and role modeling) on LMX quality and the internalization of emotional regulation. Finally, the participants were nursing staff, which contributes to difficulties in generalizing the findings to other occupation and organization types. Future studies are recommended to include participants from other occupations.

Furthermore, this study relies exclusively on a sample of female nurses. Although this gender homogeneity reflects the current demographic reality of the nursing profession in Taiwan—where females constitute the vast majority of the workforce [59]—it limits the generalizability of the findings to the entire nursing population, particularly male nurses. Prior research indicates that gender differences influence interpersonal dynamics and organizational behavior; for instance, females tend to be more interpersonally sensitive and are thus more likely to derive psychological benefits from high‐quality LMX [60]. In contrast, male employees may derive greater mental health benefits from high job autonomy than their female counterparts [61]. Consequently, the psychological mechanisms identified in this study might manifest differently in mixed‐gender or male‐dominated nursing teams. Future research is encouraged to include more gender‐diverse samples to examine the robustness of these mechanisms across different gender groups.

Finally, the measurement of satisfaction with clinical teacher teaching in this study relied on a single‐item global assessment. Although single‐item scales are highly efficient and possess strong face validity for evaluating overall attitudes or affective states, they may lack the granularity and comprehensiveness of multi‐item instruments in capturing the multidimensional nature of mentor–mentee relationships. Consequently, future research should utilize multidimensional or multi‐item measurement tools to more precisely distinguish various relationship facets, mitigate potential affective evaluation bias, and ultimately enhance the methodological rigor and explanatory power of the research findings.

### 4.2. Recommendations for Practice

The autonomy of negative emotion suppression is a key factor of physical and psychological health [15]. In Sinitic cultures such as Taiwan’s, which place great emphasis on harmony and hierarchical relationships, employees are likely to employ controlled self‐regulation, which is not conducive to their physical and mental development [15]. Hence, encouraging the internalization of emotional regulation in such cultures is critical. This study revealed the key roles of LMX in nurses’ internalization of emotional regulation, a discovery that has major implications for hospital employee management strategies. Improving the quality of LMX between nursing staff and their supervisors is an area that hospital administrators should pay attention to. In previous studies, LMX quality was primarily influenced by supervisors. Accordingly, hospitals can focus on supervisor training in areas such as contingent reward behavior, transformational leadership, and their expectations of follower success [16]. Supervisors should attempt to develop and maintain stable, positive relationships with employees. Although supervisors probably cannot treat all employees in the exactly same manner, they must at minimum make every employee feel respected and avoid making them feel like second‐class citizens [62]. In addition, hospitals can provide suitable communication channels, establish an organizational culture based on trusting relationships [63], and create a working environment in which nursing staff have a sense of belonging [64]. The quality of the LMX of nursing staff and supervisors can be effectively improved through supervisor training and improvements to the hospital environment. This would promote nurse autonomy and the regulation of negative emotions.

The study also verified that mentoring functions moderate the relationship between LMX quality and the internalization of emotional regulation. Therefore, hospitals should focus on approaches to enhance mentoring. Hospitals train new nurses through the clinical teacher system and thus devote resources to train senior nurses as clinical teachers. This training can include content on approaches to teaching and learning, conflict management, and sociability, each of which helps clarify the meaning and goals of clinical teacher roles [65]. Training can be conducted in the form of workshops, enabling teacher participants to share experience, knowledge, and skills and thus jointly develop their teaching capabilities [66]. In addition, the effectiveness of clinical teacher training should be regularly assessed and improved [67]. Enhancing the capabilities of clinical teachers and improving the performance and capabilities of new nurses positively influence LMX quality [16].

When hospitals have established a comprehensive clinical teacher system, it not only reduces the turnover rate of new nurses [43] but also increases the willingness of nursing staff who have gained from the training to become clinical teachers themselves and pass on their experience to the next generation of nursing staff [68]. The findings of Cheng et al. [69] revealed a positive correlation for the knowledge sharing behavior of mentors and apprentices. The knowledge‐sharing attitude and behavior of mentors constantly influence those of apprentices, a phenomenon that is consistent with the trickle‐down model. In the model, the specific attitudes or behaviors of higher level managers trickle down, causing lower level employees to have the same attitudes or behaviors [70]. Such mentorship, passed from generation to generation, can establish a comprehensive mentorship culture in hospitals.

## 5. Conclusion

The Taiwanese culture of collectivism and high‐power distance is not conducive to the internalization of individual emotional regulation. This study verified that if nursing staff can establish a favorable relationship with their supervisors, they experience a higher degree of emotional regulation internalization, allowing them to manage negative emotions more autonomously, which may contribute to physical and psychological health. Hospitals should also improve career development functions, psychosocial functions, and role modeling by strengthening mentoring functions as well as encouraging the guidance and assistance of clinical teachers. These efforts can help new nurses adapt to the hospital work environment, improve clinical nursing capabilities, enable the development of constructive nurse–supervisor relationships, and improve the internalization of emotional regulation, thereby nurturing competent, healthy nurses.

## Funding

This study was funded by the Ministry of Science and Technology in Taiwan (MOST 108‐2410‐H‐040‐006‐).

## Disclosure

The funders had no role in study design, data collection and analysis, decision to publish, or preparation of the manuscript.

## Conflicts of Interest

The author declares no conflicts of interest.

## Data Availability

The data that support the findings of this study are available from the corresponding author upon reasonable request.

## References

[1] Chu L. C. , The Moderating Role of Authoritarian Leadership on the Relationship Between the Internalization of Emotional Regulation and the well-being of Employees, Leadership. (2014) 10, no. 3, 326–343, 10.1177/1742715013498403, 2-s2.0-84907509792.

[2] Kumar N. , Liu Z. , Flinchbaugh C. , Hossain M. Y. , and Hossain M. N. , Impact of Emotional Labour on Taking Charge to Predict Employee’s Creative and Task Performance: the Moderation of Performance-based Pay from the Lens of self-determination Theory, PLoS One. (2022) 17, no. 10, 10.1371/journal.pone.0269196. PMC9536575 36201523

[3] Cui L. , Tang G. , and Huang M. , Expressive Suppression, Confucian Zhong Yong Thinking, and Psychosocial Adjustment Among Chinese Young Adults, Asian Journal of Social Psychology. (2022) 25, no. 4, 715–730, 10.1111/ajsp.12529.

[4] Wu M. C. , Chiang W. J. , Chiang S. L. , Trung P. M. , and Lindayani L. , A Study on Major Factors Revitalizing Nursing Staff′s Work Enthusiasm a cross-national Study on Organizational Culture, Organizational Empowerment and self-efficacy, International Journal of Healthcare Management. (2023) 16, no. 1, 93–103, 10.1080/20479700.2022.2076043.

[5] Theodosius C. , Koulouglioti C. , Kersten P. , and Rosten C. , Collegial Surface Acting Emotional Labour, Burnout and Intention to Leave in Novice and Pre‐Retirement Nurses in the United Kingdom: a Cross‐Sectional Study, Nursing Open. (2021) 8, no. 1, 463–472, 10.1002/nop2.649. 33318854 PMC7729549

[6] Xiang C. C. , Wang X. , Xie T. T. , and Fu C. L. , Differential Effects of Work and Family Support on the Relationship Between Surface Acting and Wellbeing: a self-determination Theory Approach, Psychological Reports. (2023) 126, no. 1, 198–219, 10.1177/00332941211048471. 34783268

[7] Graves L. M. and Luciano M. M. , Self-Determination at Work: Understanding the Role of leader-member Exchange, Motivation and Emotion. (2013) 37, no. 3, 518–536, 10.1007/s11031-012-9336-z, 2-s2.0-84881241714.

[8] Henderson A. A. and Jeong S. S. , Leader–Member Exchange (LMX) and Work Performance: an Application of self-determination Theory in the Work Context, European Journal of Work & Organizational Psychology. (2024) 33, no. 3, 310–324, 10.1080/1359432X.2023.2276535.

[9] Lima M. S. and Alzyood M. , The Impact of Preceptorship on the Newly Qualified Nurse and Preceptors Working in a Critical Care Environment: an Integrative Literature Review, Nursing in Critical Care. (2024) 29, no. 5, 1178–1189, 10.1111/nicc.13061. 38511618

[10] Chipwanya E. , Downing C. , and Nkosi E. , The Effect of a Preceptorship Programme on Newly Hired Experienced Professional Nurses’ self-efficacy in Nursing Clinical Competency in Saudi Arabia, International Journal African Nursing Science. (2024) 20, 10.1016/j.ijans.2024.100682.

[11] Kim Y. , Deci E. L. , and Zuckerman M. , The Development of the self-regulation of Withholding Negative Emotions Questionnaire, Educational and Psychological Measurement. (2002) 62, no. 2, 316–336, 10.1177/0013164402062002008, 2-s2.0-0036245805.

[12] Deci E. L. and Ryan R. M. , The “What” And “Why” of Goal Pursuits: Human Needs and the self-determination of Behavior, Psychological Inquiry. (2000) 11, no. 4, 227–268, 10.1207/S15327965PLI1104_01, 2-s2.0-0034549672.

[13] Deci E. L. and Ryan R. M. , Intrinsic Motivation and self-determination in Human Behavior, 2013, Springer Science & Business Media.

[14] Ryan R. M. and Connell J. P. , Perceived Locus of Causality and Internalization: Examining Reasons for Acting in Two Domains, Journal of Personality and Social Psychology. (1989) 57, no. 5, 749–761, 10.1037//0022-3514.57.5.749. 2810024

[15] Chu L. C. , The Influence of the Internalization of Emotional Regulation on Mental Health Among the Taiwanese People: the Moderating Effect of Cultural Fit, Asia Pacific Journal of Public Health. (2015) 27, no. 2, NP1918–NP1931, 10.1177/1010539512455045, 2-s2.0-84926295158. 22865723

[16] Dulebohn J. H. , Bommer W. H. , Liden R. C. , Brouer R. L. , and Ferris G. R. , A meta-analysis of Antecedents and Consequences of leader-member Exchange: Integrating the past with an Eye Toward the Future, Journal of Management. (2012) 38, no. 6, 1715–1759, 10.1177/0149206311415280, 2-s2.0-84867095839.

[17] Graen G. B. and Uhl-Bien M. , Relationship-Based Approach to Leadership: Development of leader-member Exchange (LMX) Theory of Leadership over 25 years: Applying a multi-level multi-domain Perspective, The Leadership Quarterly. (1995) 6, no. 2, 219–247, 10.1016/1048-9843(95)90036-5, 2-s2.0-58149322472.

[18] Liao S. H. and Chen C. C. , Leader-Member Exchange and Employee Creativity: Knowledge Sharing: the Moderated Mediating Role of Psychological Contract, Leadership Organ Deviation Journal. (2018) 39, no. 3, 419–435, 10.1108/LODJ-05-2017-0129, 2-s2.0-85045842261.

[19] Xie Z. , Wu N. , Yue T. , Jie J. , and Fu A. , How leader-member Exchange Affects Creative Performance: an Examination from the Perspective of self-determination Theory, Frontiers in Psychology. (2020) 11, 10.3389/fpsyg.2020.573793. PMC7655925 33192872

[20] Varma A. , Jaiswal A. , Pereira V. , and Kumar Y. L. N. , Leader-Member Exchange in the Age of Remote Work, Human Resource Development International. (2022) 25, no. 2, 219–230, 10.1080/13678868.2022.2047873.

[21] Van Strydonck I. , Decramer A. , Peccei R. , and Audenaert M. , Process and Content in Performance Management: How Consistency and Supervisor Developmental Feedback Decrease Emotional Exhaustion via high-quality LMX, Review of Public Personnel Administration. (2025) 45, no. 2, 365–398, 10.1177/0734371X231220938.

[22] Yikilmaz I. , Surucu L. , Maslakci A. , Dalmis A. B. , and Toros E. , Exploring the Relationship Between Surface Acting, Job Stress, and Emotional Exhaustion in Health Professionals: the Moderating Role of LMX, Behavioral Sciences. (2024) 14, no. 8, 10.3390/bs14080637. PMC11351417 39199033

[23] Das S. S. and Pattanayak S. , Understanding the Effect of Leadership Styles on Employee well-being Through leader-member Exchange, Current Psychology. (2023) 42, no. 25, 21310–21325, 10.1007/s12144-022-03243-3.

[24] Hanley Y. D. , Maykrantz S. A. , and Houghton J. D. , Broken Engagement: the Role of Grit and LMX in Enhancing Faculty Engagement, Higher Education Quarterly. (2024) 78, no. 1, 153–172, 10.1111/hequ.12450.

[25] Zhao T. , Li H. , Zheng L. , and Zhang Y. , How Dispositional Gratitude Shapes Employee well-being and Organizational Commitment: the Mediating Roles of Leader-Member Exchange and Coworker Exchange, Journal of Car Assess. (2023) 31, no. 1, 149–171, 10.1177/10690727221099867.

[26] Saygili M. , Hikmet N. , and Yorgancioglu Tarcan G. , The Effect of leader–member Exchange on Turnover Intention in Healthcare Employees, Journal of Health Organization and Management. (2025) 39, no. 7, 1364–1379, 10.1108/JHOM-09-2024-0394. 40032598

[27] Glasø L. and Einarsen S. , Emotion Regulation in leader–follower Relationships, European Journal of Work & Organizational Psychology. (2008) 17, no. 4, 482–500, 10.1080/13594320801994960, 2-s2.0-56049120822.

[28] Xu J. , Liu Y. , and Guo Y. , The Role of Subordinate Emotional Masking in leader–member Exchange and Outcomes: a two-sample Investigation, Journal of Business Research. (2014) 67, no. 2, 100–107, 10.1016/j.jbusres.2012.11.011, 2-s2.0-84888439525.

[29] Medler-Liraz H. and Seger-Guttmann T. , Authentic Emotional Displays, leader–member Exchange, and Emotional Exhaustion, Journal of Leadership Organ Student. (2018) 25, no. 1, 76–84, 10.1177/1548051817725266, 2-s2.0-85039912320.

[30] Gross J. J. and John O. P. , Individual Differences in Two Emotion Regulation Processes: Implications for Affect, Relationships, and well-being, Journal of Personality and Social Psychology. (2003) 85, no. 2, 348–362, 10.1037/0022-3514.85.2.348, 2-s2.0-0242474299. 12916575

[31] Ryan R. M. and Deci E. L. , Self-Determination Theory and the Facilitation of Intrinsic Motivation, Social Development, and well-being, American Psychologist. (2000) 55, no. 1, 68–78. 11392867 10.1037//0003-066x.55.1.68

[32] Leduc J. G. , Boucher F. , Marques D. L. , and Brunelle E. , Basic Psychological Need Satisfaction of Collegiate Athletes: the Unique and Interactive Effects of Team Identification and LMX Quality, Frontiers in Sports and Active Living. (2024) 6, 10.3389/fspor.2024.1342995. PMC11096468 38756189

[33] Benita M. , Arbel R. , and Milyavskaya M. , Autonomous Versus Controlled Goal Motivation Differentially Predicts Goal Progress and well-being Through Emotion Regulation Styles, Motivation Science. (2023) 9, no. 3, 229–241, 10.1037/mot0000295.

[34] Kram K. E. , Phases of the Mentor Relationship, Academic Management Journal. (1983) 26, no. 4, 608–625, 10.5465/255910.

[35] Scandura T. A. , Mentorship and Career Mobility: an Empirical Investigation, Journal of Organizational Behavior. (1992) 13, no. 2, 169–174, 10.1002/job.4030130206, 2-s2.0-84986727492.

[36] Busby K. R. , Draucker C. B. , and Reising D. L. , Exploring Mentoring and Nurse Faculty: an Integrative Review, Journal of Professional Nursing. (2022) 38, 26–39, 10.1016/j.profnurs.2021.11.006. 35042587

[37] Curtis M. B. and Taylor E. Z. , Developmental Mentoring, Affective Organizational Commitment, and Knowledge Sharing in Public Accounting Firms, Journal of Knowledge Management. (2018) 22, no. 1, 142–161, 10.1108/JKM-03-2017-0097, 2-s2.0-85044067257.

[38] Allen T. D. , Eby L. T. , Poteet M. L. , Lentz E. , and Lima L. , Career Benefits Associated with Mentoring for Protégés: a meta-analysis, Journal of Applied Psychology. (2004) 89, no. 1, 127–136, 10.1037/0021-9010.89.1.127, 2-s2.0-1142289895. 14769125

[39] Janssen S. , Van Vuuren M. , and De Jong M. D. , Informal Mentoring at Work: a Review and Suggestions for Future Research, International Journal of Management Reviews. (2016) 18, no. 4, 498–517, 10.1111/ijmr.12069, 2-s2.0-84930359018.

[40] Hautala K. T. , Saylor C. R. , and O’Leary-Kelley C. , Nurses’ Perceptions of Stress and Support in the Preceptor Role, Journal of Nurses. (2007) 23, no. 2, 64–70, 10.1097/01.NND.0000266611.78315.08, 2-s2.0-34247244223. 17414854

[41] Powers K. , Herron E. K. , and Pagel J. , Nurse Preceptor Role in New Graduate Nurses’ Transition to Practice, Dimensions of Critical Care Nursing. (2019) 38, no. 3, 131–136, 10.1097/DCC.0000000000000354, 2-s2.0-85064239552. 30946120

[42] Choi E. and Yu S. , Effects of Preceptors’ Mentoring Function on Novice Nurses’ self-efficacy and Organizational Commitment: a cross-sectional Study, Nurse Education in Practice. (2022) 64, 10.1016/j.nepr.2022.103431. 36049395

[43] Zhang Y. P. , Huang X. , Xu S. Y. , Xu C. J. , Feng X. Q. , and Jin J. F. , Can a one-on-one Mentorship Program Reduce the Turnover Rate of New Graduate Nurses in China? A Longitudinal Study, Nurse Education in Practice. (2019) 40, 10.1016/j.nepr.2019.08.010, 2-s2.0-85071930810. 31518894

[44] Podsakoff P. M. , MacKenzie S. B. , Lee J. Y. , and Podsakoff N. P. , Common Method Biases in Behavioral Research: a Critical Review of the Literature and Recommended Remedies, Journal of Applied Psychology. (2003) 88, no. 5, 879–903, 10.1037/0021-9010.88.5.879, 2-s2.0-0141907688. 14516251

[45] Tabachnick B. G. , Fidell L. S. , and Ullman J. B. , Using Multivariate Statistics, 2007, 5th edition, Pearson.

[46] Liden R. C. and Maslyn J. M. , Multidimensionality of leader-member Exchange: an Empirical Assessment Through Scale Development, Journal of Management. (1998) 24, no. 1, 43–72, 10.1016/S0149-2063(99)80053-1.

[47] Huang I. C. , Du P. L. , Wu L. F. , Achyldurdyyeva J. , Wu L. C. , and Lin C. S. , Leader–Member Exchange, Employee Turnover Intention and Presenteeism: the Mediating Role of Perceived Organizational Support, Leadership Organ Deviation Journal. (2021) 42, no. 2, 249–264, 10.1108/LODJ-03-2020-0094.

[48] Castro S. L. , The Tale of Two Measures: Evaluation and Comparison of Scandura’s (1992) and Ragins and Mcfarlin’s (1990) Mentoring Measures, 2004, InSouthern Management Association Meeting.

[49] Chang H. C. and Uen J. F. , Shaping Organizational Citizenship Behavior of New Employees: Effects of Mentoring Functions and Supervisor Need for Achievement, Sage Open. (2022) 12, no. 1, 10.1177/21582440211068515.

[50] Amini K. , Negarandeh R. , Ramezani‐Badr F. , Moosaeifard M. , and Fallah R. , Nurses’ Autonomy Level in Teaching Hospitals and Its Relationship with the Underlying Factors, International Journal of Nursing Practice. (2015) 21, no. 1, 52–59, 10.1111/ijn.12210, 2-s2.0-84921023390. 24256084

[51] Battistelli A. , Galletta M. , Vandenberghe C. , and Odoardi C. , Perceived Organisational Support, Organisational Commitment and Self‐Competence Among Nurses: a Study in Two Italian Hospitals, Journal of Nursing Management. (2016) 24, no. 1, E44–E53, 10.1111/jonm.12287, 2-s2.0-84956672100. 25652882

[52] De Gieter S. , Hofmans J. , and Pepermans R. , Revisiting the Impact of Job Satisfaction and Organizational Commitment on Nurse Turnover Intention: an Individual Differences Analysis, International Journal of Nursing Studies. (2011) 48, no. 12, 1562–1569, 10.1016/j.ijnurstu.2011.06.007, 2-s2.0-81855205005. 21821254

[53] Vansteenkiste M. , Niemiec C. P. , and Soenens B. , The Development of the Five mini-theories of self-determination Theory: an Historical Overview, Emerging Trends, and Future Directions, The Decade Ahead: Theoretical Perspectives on Motivation and Achievement. (2010) 105–165, 10.1108/S0749-7423.

[54] Nisbett R. E. and Wilson T. D. , The Halo Effect: Evidence for Unconscious Alteration of Judgments, Journal of Personality and Social Psychology. (1977) 35, no. 4, 250–256, 10.1037/0022-3514.35.4.250.

[55] Amir A. , Shtudiner Z. , and Shavit T. , Is Happiness for All? the Happiness Halo Effect on Coworkers’ Perceptions, Frontiers in Psychology. (2025) 16, 10.3389/fpsyg.2025.1653843. PMC12815774 41567448

[56] Xu X. and Payne S. C. , Quantity, Quality, and Satisfaction with Mentoring: what Matters Most?, Journal of Career Development. (2014) 41, no. 6, 507–525, 10.1177/0894845313515946, 2-s2.0-84927643892.

[57] Galletta M. , Portoghese I. , and Battistelli A. , Intrinsic Motivation, Job Autonomy and Turnover Intention in the Italian Healthcare: the Mediating Role of Affective Commitment, Journal of Management Research. (2011) 3, no. 2, 1–9, 10.5296/jmr.v3i2.619.

[58] Salami S. O. , Mentoring and Work Attitudes Among Nurses: the Moderator Roles of Gender and Social Support, Europe’s Journal of Psychology. (2010) 6, no. 1, 102–126, 10.5964/ejop.v6i1.174.

[59] Hsu C. E. , Lei H. I. , and Han D. Y. , Current State and Trends of Gender Differences Among Health Care Personnel in Taiwan, Journal of Quality Healthcare. (2023) 17, no. 3, 78–83, 10.53106/199457952023051703012.

[60] Di Milia L. and Jiang Z. , Linking leader-member Exchange and work–nonwork Balance: the Mediating Role of Thriving at Work and the Moderating Role of Gender, Personnel Review. (2024) 53, no. 1, 155–172, 10.1108/PR-03-2022-0211.

[61] Lu Z. , Wang S. , Li Y. , Liu X. , and Olsen W. , Who Gains Mental Health Benefits from Work Autonomy? the Roles of Gender and Occupational Class, Applied Research in Quality of Life. (2023) 18, no. 4, 1761–1783, 10.1007/s11482-023-10161-4. PMC9990038 37359222

[62] Atitumpong A. and Badir Y. F. , Leader-Member Exchange, Learning Orientation and Innovative Work Behavior, Journal of Workplace Learning. (2018) 30, no. 1, 32–47, 10.1108/JWL-01-2017-0005, 2-s2.0-85048808319.

[63] Lee E. K. and Ji E. J. , The Moderating Role of leader–member Exchange in the Relationships Between Emotional Labor and Burnout in Clinical Nurses, Asian Nursing Research. (2018) 12, no. 1, 56–61, 10.1016/j.anr.2018.02.002, 2-s2.0-85044332858. 29463479

[64] Kim M. H. and Yi Y. J. , Impact of Leader‐Member‐Exchange and Team‐Member‐Exchange on Nurses’ Job Satisfaction and Turnover Intention, International Nursing Review. (2019) 66, no. 2, 242–249, 10.1111/inr.12491, 2-s2.0-85057292819. 30474113

[65] Hong K. J. and Yoon H. J. , Effect of Nurses’ Preceptorship Experience in Educating New Graduate Nurses and Preceptor Training Courses on Clinical Teaching Behavior, International Journal of Environmental Research Public Health. (2021) 18, no. 3, 10.3390/ijerph18030975. PMC7908293 33499327

[66] Liu L. , Fillipucci D. , and Mahajan S. M. , Quantitative Analyses of the Effectiveness of a Newly Designed Preceptor Workshop, Journal of Nurses. (2019) 35, no. 3, 144–151, 10.1097/NND.0000000000000528, 2-s2.0-85065525044. 30762844

[67] Odelius A. , Traynor M. , Mehigan S. , Wasike M. , and Caldwell C. , Implementing and Assessing the Value of Nursing Preceptorship, Nursing Management. (2017) 23, no. 9, 35–37, 10.7748/nm.2017.e1547, 2-s2.0-85012049698. 28132615

[68] McBride A. B. , Campbell J. , and Deming K. , Does Having Been Mentored Affect Subsequent Mentoring?, Journal of Professional Nursing. (2019) 35, no. 3, 156–161, 10.1016/j.profnurs.2018.11.003, 2-s2.0-85058164849. 31126390

[69] Cheng Y. N. , Hu C. , and Chien S. W. , Mentor Functions and Protégé Knowledge Sharing, International Journal Strategy. (2013) 5, no. 1, 37–56.

[70] Wo D. X. , Ambrose M. L. , and Schminke M. , What Drives trickle-down Effects? A Test of Multiple Mediation Processes, Academy of Management Journal. (2015) 58, no. 6, 1848–1868, 10.5465/amj.2013.0670, 2-s2.0-84975764301.

